# Effect of Insecticide Regimens on Biological Control of the Tarnished Plant Bug, *Lygus lineolaris*, by *Peristenus* spp. in New York State Apple Orchards

**DOI:** 10.1673/031.010.3601

**Published:** 2010-04-17

**Authors:** Lora A. Crampton, Greg M. Loeb, Kim A. Hoelmer, Michael P. Hoffmann

**Affiliations:** ^1^ Department of Entomology, Cornell University, Ithaca, New York. 14853; ^2^ Department of Entomology, Cornell University, New York State Agricultural Experiment Station, Geneva, NY 14456; ^3^ USDA ARS, Beneficial Insects Introduction Research Unit, Newark, DE 19713

**Keywords:** Reduced risk, organic, natural enemies, beneficial insects

## Abstract

To improve biological control of *Lygus lineolaris* (Palisot de Beauvois) (Hemiptera: Miridae), the European parasitoid *Peristenus digoneutis* Loan (Hymenoptera: Braconidae) was introduced into the US in the 1980's and has become established in forage alfalfa, strawberries and apples. The objective of this study was to determine how four different insecticide management regimes affected parasitism of *L. lineolaris* by *Peristenus* spp. During the summers of 2005 and 2006, *L. lineolaris* nymphs were collected from New York State apple orchards using industry standard, reduced risk, and organically approved insecticides only. A ‘no insecticide’ (abandoned orchard) treatment was also included in 2006. Rates of parasitism of *L. lineolaris* nymphs were determined using a DNA-based laboratory technique. Results indicated that insecticide treatment had a significant effect on rates of parasitism of *L. lineolaris* by *Peristenus* spp. Compared to the industry standard treatment, rates of parasitism were higher in reduced risk orchards and lower in organic orchards. These results suggest that it is difficult to predict *a priori* the consequences of insecticide programs and point to the need to take into consideration the specific pests and beneficial organisms involved as well as the crop and the specific insecticides being applied.

## Introduction

Apple producers in New York State and the northeastern United States are challenged by a formidable array of insect pests and diseases (Agnello et al. 2006). The intense disease and arthropod pressures, combined with the unpredictable and sometimes severe weather of the northeastern United States, create difficult growing conditions for New York State apple producers.

To control insect pests, apple growers utilize a range of chemical regimens. Standard growing practices rely heavily on broad-spectrum organophosphate, carbamate, and pyrethroid insecticides (Agnello 2007). These insecticides have long caused concern because of risks to humans, the environment and beneficial arthropods (Eskenazi and Maizlish 1988; Rosenstock et al. 1991; Van Driesche and Bellows 1996; Ruberson et al. 1998; Beane Freeman et al. 2005). Scientific and public concern about organophosphate, carbamate and pyrethroid insecticides led to passage of the Food Quality Protection Act in 1996 (Public Law 104–170) which calls for an eventual elimination of many of these insecticides from use in the United States.

In response to the eventual loss or restricted use of organophosphate, carbamate and pyrethroid insecticides, the Reduced-Risk Management Program, a collaborative effort consisting of scientists, growers, representatives from the pesticide industry, and the U.S. Department of Agriculture, was formed to evaluate newer reduced risk insecticides under realistic field conditions. Reduced risk insecticides have been marketed as good alternative products because they are purported to be safer for the environment and more pest specific and they conform to the Food Quality Protection Act (Balazs et al. 1997; Pekar 1999; Hill and Foster 2003). Reduced risk insecticides labeled for use on apples include Apollo® (clofentizine), Nexter® (pyridaben), Zeal® (etoxazole), Actara® (thiamethoxam), Esteem® (pyriproxyfen), Avaunt® (indoxacarb), Assail® (acetamiprid), Intrepid® (methoxyfenozide), SpinTor® (spinosad) and dormant oil (petroleum oil). Although frequently found to be more selective than older generation insecticides (e.g. Atanassov et al. 2003; Musser and Shelton 2003; Koss et al. 2005), research into indoxacarb (Haseeb et al. 2004), imidacloprid, thiamethoxam, acetamiprid (Williams et al. 2003; Nasreen et al. 2004; Beers et al. 2005; Poletti et al. 2007) and spinosad (Nowak et al. 2001; Cisneros et al. 2002; Schneider et al. 2003) have shown them to be toxic to some beneficial arthropods (also see Stark and Banks 2001; Brunner et al. 2001). In addition, many reduced risk chemicals are significantly more expensive than standard products.

Another treatment option is producing apples under an organic regimen. While there are few large organic apple producers in New York State, there is increasing interest from growers and consumers in the burgeoning organic market across the U.S. (Peck et al. 2005). Organic apples receive a premium at market but organic growers are limited in what can be applied for arthropod and disease control. Certified organic producers can utilize non-synthetic, naturally derived compounds made from botanicals, elemental compounds (e.g. sulfur and copper), and physical barriers. However, many of the organic compounds are more expensive than, and not as effective as, their synthetic counterparts.

As the Food Quality Protection Act is implemented, the increased cost of reduced risk insecticides will weigh heavily on apple growers. This creates an immediate need for research into alternatives that may reduce the need for insecticide sprays, such as biological control. In addition, research is needed to clarify how new reduced risk insecticides affect non target species, including natural enemies, compared to conventional, more broad-spectrum insecticides or pesticides allowed in organic apple production in the Northeast.

The tarnished plant bug, *Lygus lineolaris* (Palisot de Beauvois) (Hemiptera: Miridae), is an important direct pest of apples and many other crops. In apples it causes damage by feeding on the developing flowers and fruit, resulting in abscission of flower buds, fruit underdevelopment, and malformations (Prokopy and Hubbell 1981). Abscission of buds is of no economic consequence; however, underdeveloped fruit and malformations can result in culling and downgrading (Weires et al. 1985; Michaud et al. 1989). Fruit malformations from *L. lineolaris* can be characterized as puncture wounds, scabs or “cat facing” (Boivin and Stewart 1983a).

There are several native species of parasitoids (Hymenoptera: Braconidae) in the *Peristenus pallipes* species complex and two introduced European species, *P. digoneutis* Loan and *P. rubricollis* (Thomson) that parasitize nymphs of *L. lineolaris* (Goulet and Mason 2006). According to Day et al. (1990) the native species complex was not able to provide adequate biological control of *L. lineolaris* and therefore *P. digoneutis* was introduced from France in the 1980's. After introduction into the U.S., robust populations of *P. digoneutis* developed in forage alfalfa, reducing the total *L. lineolaris* population in that crop by 75% (Day 2005). As expected, *P. digoneutis* not only expanded its geographic range but also spread from alfalfa fields to high value crops such as strawberries (Tilmon and Hoffmann 2002) and apples (Crampton 2007). *Peristenus rubricollis* is only occasionally found parasitizing *L. lineolaris*, preferring other mirids in the *Adelphocoris* genus, especially the introduced European alfalfa plant bug, *A. lineolatus* (Goeze), which it was introduced to control (Day 2005).

The objective of this study was to examine the effect of pesticide regimen on rates of parasitism by *Peristenus* spp. on *L. lineolaris* in apple orchards. We specifically compared parasitism in commercial apples produced using the industry standard insecticide regime, organically approved insecticides only, and reduced risk insecticides. We also included abandoned apple orchards where no insecticides were applied.

## Materials and Methods

### Regimens

During this field study, conducted in the summers of 2005 and 2006, *L. lineolaris* nymphs were collected from apple orchards under four different arthropod management regimens: organic, standard reduced risk and abandoned. Standard insecticide regimens are defined here as the current convention in apple production, which relies heavily on organophosphate, carbamate, and pyrethroid insecticides. The reduced risk regimen (defined above) included the following insecticides (active ingredients) clofentizine, pyridaben, etoxazole, thiamethoxam, pyriproxyfen, indoxacarb, acetamiprid, benzoic acid, spinosad and petroleum oil (see Agnello et al. 2004 and Sarvary et al. 2007 for complete lists of pesticides used in reduced risk and standard orchards). Reduced risk regimens were only available in 2005. The organic orchards selected were certified organic and followed guidelines mandated by the U.S. Department of Agriculture National Organic Program (Agricultural Marketing Service, 2000). Insecticides used in organic orchards included sulfur and kaolin clay. The abandoned orchards were typically no longer under any form of management and consequently had excessive weed populations and high infestations and damage by both insect pests and disease.

### Orchards

**Figure 1.**
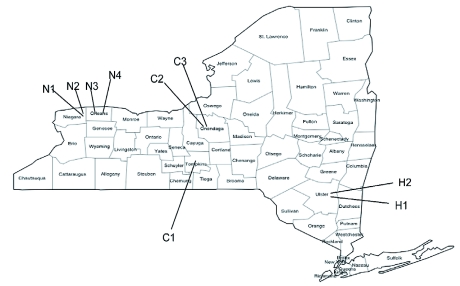
Location of apple orchards in New York State used in this study. Explanation of orchard abbreviations provided in Table 1. High quality figures are available online.

The field sites were located within nine commercial orchards in three of the major apple producing regions of New York State Hudson Valley, Central and Niagara (Table 1 and Figure 1). Six of the nine orchards were participants in the Reduced-Risk Management Program (2005 only). At these locations, two approximately 10 acre blocks were under either the reduced risk or standard treatment regimen in a split plot design. Organic plots in the same regions were selected to compliment the split plot design. At farm N2, two organic plots were selected, labeled organic(P) and organic(G). The plot organic(G) was only available in 2005. In the Niagara region and Hudson Valley regions, organic plots were very close to the corresponding reduced risk/standard plots, sharing a common property line. However, the organic plot in central New York State (farm C1) was 45 miles from the closest reduced risk/standard plots (C2) (Figure 1). Abandoned plots were also selected from the same area. Two shared a property line with reduced risk/ standard plots and the third was within 5 miles from the organic/standard plots (farm N2).

**Table 1.**
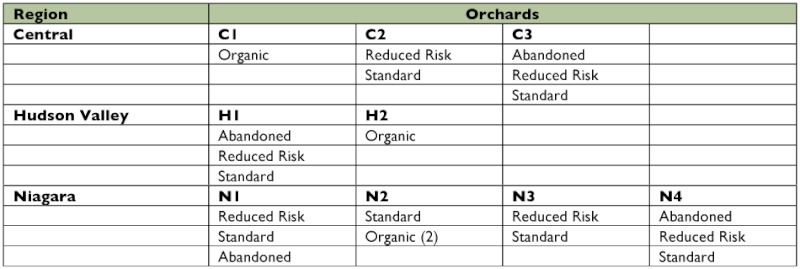
Insecticide regimens from the nine orchards used in this study. Note that Reduced Risk orchards were only available in 2005.

### Field Sampling

*Lygus lineolaris* nymph samples were collected weekly during June, July and August in 2005, except when prevented by inclement weather or pesticide re-entry restrictions. In 2006, samples were taken on four separate weeks during the season to correspond to parasitism levels based on sampling results from 2005. A sweep net was used to sample for *L. lineolaris* nymphs in ground cover in an area approximately 0.40 ha in size, located in the middle of each treatment block of apples. Sweeping of the orchard ground cover continued until 24 to 30 *L. lineolaris* nymphs were collected or 2.5 hours passed. Ground cover was similar between regimens within orchards, with the exception of organic(P) treatment at farm N2. Typical ground cover consisted of *Dactylis* spp., *Trifolium* spp., *Vicia* spp., *Hieracium* spp., *Plantago* spp., *Taraxacum* spp., *Leontodon* spp., *Galium* spp., and grass. In organic(P) *Galium* spp. constituted about 80% of the ground vegetation. Nymphs collected by sweep net were preserved in 95% ethanol, and stored on ice until transferred to a -20°C freezer.

### Determining adult population size

There is evidence of a weak density-dependent relationship between numbers of *L. lineolaris* nymphs and *P. digoneutis* (Tilmon 2001; Day 2005). A density-dependent relationship is important in this study because if one of the regimens had significantly more nymphs than the other regimens the interpretation of the parasitism rates would change. Since adult and nymphal population densities are related (Day 2005), relative adult densities were estimated using yellow sticky cards (Prokopy and Hubbell 1981; Boivin & Stewart 1982; Boivin and Stewart 1983b). A row within the 0.40 ha *L. lineolaris* collection area was arbitrarily chosen at the beginning of the study and each week thereafter five 13.5 cm × 13.5 cm sticky cards, spaced 50 meters apart, were hung in trees approximately 0.75 meters above the ground. Cards were replaced each time the site was visited, wrapped in cellophane, and stored at 4°C until examined for *L. lineolaris* adults.

### Determining Parasitism Rates

The molecular identification of *Peristenus* spp. was achieved using a technique developed by Tilmon et al. (2000). Briefly, DNA was extracted from each *L. lineolaris* nymph and possible parasitoid larva contained within them. Polymerase Chain Reaction (PCR) primers specific to *L. lineolaris* were amplified, thereby confirming success of the extractions. Finally, another PCR with specific primers amplified *Peristenus* spp. genes that allowed parasitized *L. lineolaris* nymphs to be differentiated from unparasitized nymphs. Samples were stored at 20° C. DNA was extracted using DNAzol, (Invitrogen, www.invitrogen.com) following the manufacture's protocol, except the ‘Lysis step’ was scaled down from 1ml to 100 µl. Primers C1-J-2252, C1-J-2183 and TL2-N3014 were obtained from Integrated DNA Technologies (Coralville, IA). Taq polymerase was obtained from Promega (www.promega.com). PCR conditions as outlined by Tilmon et al. (2000) were followed.

### Statistical Analysis

A General Linear Model with a log link and negative binomial distribution was used to describe the adult *L. lineolaris* count number and test for treatment effects. Orchards were considered a repeated measure. The negative binomial distribution was selected instead of a Poisson distribution because the assumption of variance equaling mean was violated, indicating over dispersion. Variance in the negative binomial distribution is a dispersion parameter estimated by maximum likelihood.

**Figure 2.**
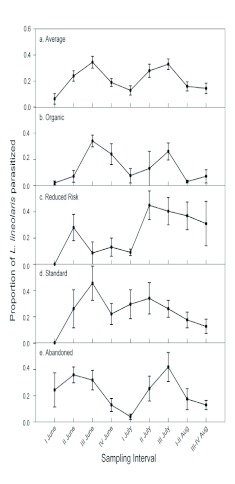
Mean parasitism, ± standard error, of *Lygus lineolaris* by *Peristenus* spp for a-all orchards combined; b- organic; creduced risk; d- standard; and e- abandoned orchards over time. All values represent data combined from 2005–2006. High quality figures are available online.

Another General Linear Model with a logit link and a binomial distribution regression was used to analyze the relevance of treatment on parasitism of *L. lineolaris* by *Peristenus* spp. Again, orchards were considered a repeated measure. Since it is well established that there are two seasonal peaks in parasitism because *P. digoneutis* is bivoltine (Day et al. 1992; Tilmon 2001; Goulet and Mason 2006) (also see Figure 2), time was coded as a categorical value. This approach allows the magnitude of parasitism to change over the season. For the purpose of this study total parasitism (all *Peristenus* species combined) was used, thereby creating a dichotomous response variable, (i.e. parasitized, not parasitized). Furthermore, for analyzing the effect of pesticide treatment on parasitism, *Peristenus* spp. was the unit of interest. All statistical analyses were preformed using SAS 9.1(2003).

## Results

Parasitism rates of *L. lineolaris* nymphs by *Peristenus* species, averaged over the different treatments and regions, indicate two distinct peaks occurring in mid-June and mid-July (Figure 2a). The overall parasitism rates, averaged across time and orchards, ranged from 14% in organic orchards in the Niagara region to a high of 37% in an abandoned orchard in the central New York State region (Table 2). The pattern of parasitism over the season was roughly similar among pesticide regimes (peaks in June and July), although the magnitude of peak levels differed, as well as the width of the peaks (Figure 2b–e). Reduced risk orchards stood out in that peak rates were higher in July compared to June and remained high well into August.

The logistic model for parasitism indicated that insecticide treatment was a significant predictor of parasitism (_χ_2 = 33.1, df = 3, P < 0.0001). Total parasitism in standard regimens was not significantly different from that in abandoned orchards (P = 0.1063). However, organic farms had significantly lower likelihood of parasitism when compared with standard (*P* < 0.0001). The odds ratio indicates that the likelihood of encountering a parasitized *L. lineolaris* nymph in an organic orchard is 0.606 times that of encountering a parasitized nymph in a standard orchard (39.4%) less likely to be parasitized) (See Table 3). Reduced risk orchards had significantly higher likelihood of parasitism than standard orchards (*P* = 0.0413). The odds ratio indicates that a *L. lineolaris* nymph in a reduced risk orchard is 1.258 times or 25.8% more likely to be parasitized than a nymph in a standard orchard (Table 3).

**Table 2.**
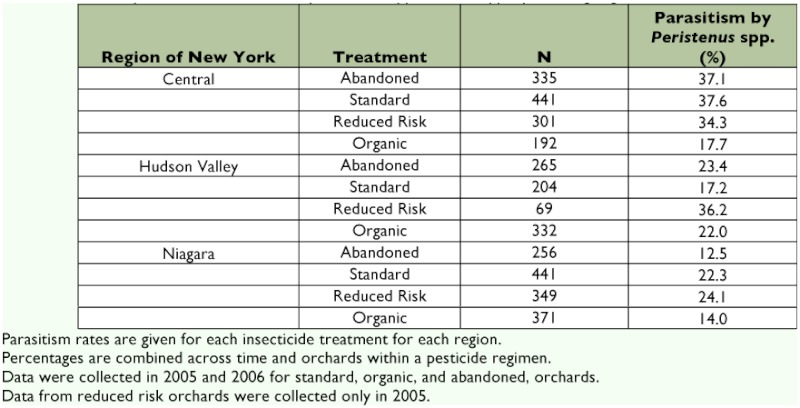
Percent parasitism of *L. lineolaris* by *Peristenus* spp. in three apple producing regions of New York State.

**Table 3.**
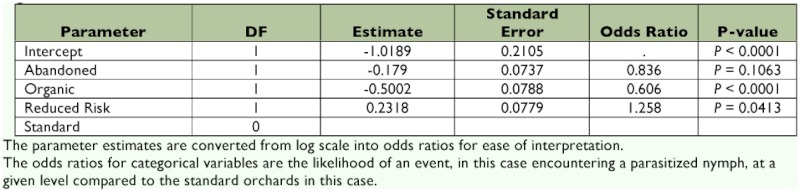
Logistic regression of *Peristenus* spp. parasitism of *L. lineolaris* nymphs in apple orchards under different pesticide regimens.

The Pearson _χ_^2^ squared test indicated the negative binomial regression model was an adequate fit for adult abundance on sticky cards (_χ_^2^ = 1055.6, df = 881; _χ_^2^ / df = 1.2). The analysis indicates that pesticide treatment was a significant predictor of adult *L. lineolaris* count (_χ_^2^ = 35.99, df = 3, P < 0.0001) (Table 4). There was no statistically significant difference between the numbers of *L. lineolaris* in standard and reduced risk orchards (P = 0.1518). There were significant differences between organic and standard (P < 0.0001) and between abandoned and standard (P = 0.0015). The positive abandoned estimate indicates that abandoned orchards had more *L. lineolaris* adults than standard orchards. Organic orchards also had more *L. lineolaris* than standard orchards, which is described by the positive organic parameter estimate (see Table 4).

## Discussion

An intriguing result of this study is that there was no significant difference in parasitism between the abandoned orchards and the standard orchards. This indicates that the *Peristenus* spp. parasitoids were able to attack *L. lineolaris* nymph hosts in the standard insecticide environment as effectively as in the abandoned environment where no insecticides were applied. In fact, given the density dependent relationship between parasitism and nymph populations and the greater numbers of adult *L. lineolaris* in abandoned orchards compared to standard orchards, it suggests parasitism rates were effectively greater in standard orchards than abandoned orchards. Reasons for this difference are unclear. One possible explanation is that the environment (e.g., higher plant diversity) within abandoned orchards is more favorable for *L. lineolaris* than for *Peristenus*. However, additional experiments are necessary to explain this result.

The regression comparison between standard and reduced risk insecticide regimens was significant; a nymph encountered in a reduced risk orchard was 25.8% more likely to be parasitized than a nymph in a standard orchard. This result is bolstered by the relative adult density data that indicated density of *L. lineolaris* in reduced risk and standard orchards was not significantly different. This study indicates that, in the case of *Peristenus* spp, the reduced risk regimen has a less disruptive effect on biological control than the standard insecticide regimen. This result is consistent with other field studies comparing the impact of different pesticide regimes on beneficial arthropods (Balazs et al. 1997; Pekar 1999; Atanassov et al. 2003; Hill and Foster 2003; Musser and Shelton 2003; Koss et al. 2005). Some field studies, however, have found no differences in natural enemy abundance or impact between conventional and reduced-risk regimes, indicating results are likely to be crop and natural enemy dependent (Jenkins and Isaacs 2007; Sarvary et al. 2007).

**Table 4.**
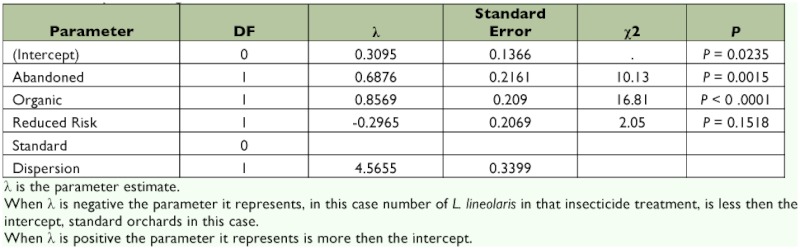
General linear model with log link and negative binomial distribution of adult *L. lineolaris* numbers in apple orchards under different pesticide regimens.

An interesting result in this study is that a nymph encountered in an organic orchard was 39.4% less likely to be parasitized than a nymph encountered in a standard orchard. Organic orchards had significantly higher *L. lineolaris* densities relative to standard orchards and, with putative positive density dependent parasitism ((Tilmon 2001; Day 2005), higher parasitism in organic orchards would have been expected than for conventional orchards. However, the opposite occurred. It is possible that organic insecticides were less lethal to *L. lineolaris*, but perhaps more lethal to the *Peristenus* spp. This could result in higher levels of *L. lineolaris* and lower rates of parasitism in the organic orchards. Clearly more research would be needed to fully understand the relationships across treatments.

While this study indicates that rates of parasitism are significantly higher in standard orchards when compared to organic apple orchards, Tilmon and Hoffmann (2002) found the opposite result for parasitism rates of *L. lineolaris* by *Peristenus* in strawberries. In their study, *Peristenus* parasitism was 5–6.5 times more likely in an organic strawberry field than in a conventional (standard) field. A key difference between these studies is that the organic strawberry fields sampled did not have any pesticides applied, whereas all of the organic apple orchards applied USDA-certified organic pesticides. This suggests that the application of organic pesticides can have a negative impact on *Peristenus* spp. Furthermore, a model by Kovach et al. (1992) of apple production in New York State suggested that the environmental impact of organic apple production was greater than that of standard apple production. This evidence suggests that the lower likelihood of parasitism by *Peristenus* spp. in organic orchards was due to organic pesticides; however, further research is required to establish cause and effect relationships of the organic pesticides on beneficial arthropods.

The overall conclusion from this two-year project is that pesticide regimes in apples have significant influence on biological control of *L. lineolaris* by its parasitoids, although not necessarily in predicted ways. We anticipated that reduced-risk insecticides would result in increased parasitism by *Peristenus* spp. relative to conventional, more broad-spectrum insecticides, and this is what we found. Contrary to expectation, however, parasitism rates in organic orchards were lower than conventionally treated orchards. Hence, this suggests that it is difficult to predict *a priori* the consequences of insecticide programs that points to the need to take into consideration the specific pests and beneficial organisms involved as well as the crop and the specific insecticides being applied.
